# Towards the Biological Understanding of CTC: Capture Technologies, Definitions and Potential to Create Metastasis

**DOI:** 10.3390/cancers5041619

**Published:** 2013-12-04

**Authors:** Ana M.C. Barradas, Leon W.M.M. Terstappen

**Affiliations:** Department of Medical Cell Biophysics, MIRA Institute for Biomedical Technology and Technical Medicine, University of Twente, PO Box 217, Enschede 7500AE, The Netherlands; E-Mail: a.m.c.barradas@utwente.nl

**Keywords:** circulating tumor cells, image analysis, filtration, EpCAM, cancer stem cell, tumorogenesis

## Abstract

Circulating Tumor Cells (CTC) are rare cells originated from tumors that travel into the blood stream, extravasate to different organs of which only a small fraction will develop into metastasis. The presence of CTC enumerated with the CellSearch system is associated with a relative short survival and their continued presence after the first cycles of therapy indicates a futile therapy in patients with metastatic carcinomas. Detailed characterization of CTC holds the promise to enable the choice of the optimal therapy for the individual patients during the course of the disease. The phenotype, physical and biological properties are however not well understood making it difficult to assess the merit of recent technological advancements to improve upon the capture of CTC or to evaluate their metastatic potential. Here we will discuss the recent advances in the classification of CTC captured by the CellSearch system, the implications of their features and numbers. Latest capture platforms are reviewed and placed in the light of technology improvements needed to detect CTC. Physical properties, phenotype, viability and proliferative potential and means to assess their proliferation and metastatic capacity will be summarized and placed in the context of the latest CTC capture platforms.

## 1. Introduction

Circulating tumor cells (CTC) detach from tumors and enter the blood stream, eventually forming new metastasis at distant sites [1]. Their frequency is related with progression-free and overall survival in breast [2], colorectal [3], castration-resistant prostate [4], small [5,6] and non-small cell lung cancer (NSCLC) patients [7]. Availability of a sufficient number of tumor cells offers the opportunity to evaluate the presence of treatment targets and as such CTC can be used as a “liquid biopsy”. In breast cancer, CTC are a better way to predict therapy success than imaging techniques [8] and compared to locally collected tumor samples, they might better represent the tumor progression as these are the evading cancer cells. Furthermore, a blood sample analysis is faster, easier and less invasive than collecting a tissue biopsy. CTC are however extremely rare, with a frequency of typically 1–10 CTC among 6 × 10^6^ leukocytes, 2 × 10^8^ platelets and 4 × 10^9^ erythrocytes per mL of blood [9,10]. This low frequency creates tremendous technological challenges for their detection. Currently, the only FDA cleared system for CTC detection is the CellSearch system (Veridex, LLC, Raritan, NJ, USA). Recent reports describe the development of new CTC capture platforms [11,12,13,14,15,16,17] demonstrating an expanding interest in this field and attempting to circumvent the limitations of CellSearch. In this review we will provide an overview of recent CTC capture platforms, their associated CTC definition and discuss in more detail filtration based CTC enrichment, since the majority of others has been extensively reviewed elsewhere [18,19,20,21]. In the second part of this review we will address the current knowledge on CTC biological properties, with emphasis on viability, proliferative capacities and stem-cell like properties and finally we will discuss current strategies for CTC culturing.

## 2. CTC Definitions and Capture

The current CellSearch CTC definition comprises an object that stains positively for cytokeratins (CKs) 8, 18 and 19, does not stain for CD45, has a nucleus (positive 4',6-diamidino-2-phenylindole (DAPI) staining), is more than 4 × 4 μm^2^ in size and possesses a cell like morphology [9]. However, the lack of “one” CTC definition among different technologies raises the questions of (i) what are CTC, (ii) how to define them and (iii) what are the absolute minimum requirements of a new technology for users to safely rely on its ability to capture CTC? Table 1 summarizes technologies for CTC detection split over 3 sub-tables, each one comprising a different capturing method, based on physical-, biological- and both physical and biological- CTC properties. Columns 1 to 6 identify the parameters used for CTC identification (and intrinsically defining a CTC) and columns 7 to 10 provide information over the assay. Table 1 does not provide an exhaustive overview of all parameters used for CTC identification and definition in each case, but rather illustrates the discrepancies that exist among different technologies regarding the most common parameters. For this purpose, we analyzed whether the authors specifically stated to have considered expression of EpCAM (either to capture or stain after capturing), CKs, CD45, as well as presence of nucleus, specific morphology and size range. In some studies, some of these parameters were mentioned as having been tested, but it was not clear to which extend they were accounted for in the definition, being marked in the Table as NC (not clear). Following assay parameters were analyzed: numerical aperture of the microscope if CTC identification was image-based; inclusion of blind users to score objects as CTC (marked as blind); use of a threshold (or cut off value), which refers to maximum number of positive objects that are identified as CTC but not sufficient to account for a positive sample, and inclusion of healthy volunteers to test the identification method in parallel with the patient samples. For instance, whereas the CellSearch and HB-Chip require positive Epithelial Cell Adhesion Molecule (EpCAM), CKs and nuclei staining to count a cell as a CTC, the MagSweeper only requires EpCAM. Furthermore morphology is poorly accounted for, and decisive criteria such as size and shape, although mentioned in some reports, are not always specified. A threshold is often defined based on values obtained from healthy donor samples, but to determine it reliably is challenging. In the prospective clinical studies that lead to the FDA clearance of CellSearch for metastatic breast [2] and prostate cancer [4] a discrimination between patients with favorable CTC (<5/7.5 mL of blood) and unfavorable CTC (>5/7.5 mL of blood) was used and for colorectal cancer [3] <3 CTC/7.5 mL and >3 CTC/7.5 mL was made. Although this permits a simple discrimination between patients with favorable and unfavorable prognosis in actuality the chances for longer survival decreases with increasing CTC load [22,23]. The change from unfavorable to favorable CTC after the first cycles of a new therapy is associated with increased survival benefit [4,23,24] making CTC an excellent tool to monitor the effectiveness of therapy. Question remains what time period is needed to assess a meaningful decrease in CTC and how to judge what represents a decrease in CTC especially at low CTC numbers. Guidelines for the interpretation of true changes in CTC numbers have been introduced recently [22]. Bottom line however, is that the best survival prospects are when zero CTC are detected by the CellSearch system [22]. 

Given that only ~70% of breast cancer patients with metastatic disease have 1 or more CTC in 7.5 mL of blood it was surprising to see that ~20% of early breast cancer patients before surgical intervention had 1 or more CTC in 7.5 to 30 mL of blood and that the presence of CTC was associated with a significant increase in the risk of recurrence and shorter overall survival [25,26,27,28]. The sensitivity and specificity of the technology for CTC detection will need to be significantly improved before it can be reliably introduced in the early cancer disease setting. Application of a metastatic cascade model to women with breast cancer suggests that recurrence of breast cancer could be reduced from the current 9% to 1% when a CTC concentration of 9 CTC/L blood could be reliably detected [29]. Analysis of larger blood volume can be achieved through leukapheresis and a recent report indeed showed a larger number of CTC as well as a larger proportion of patients in which CTC are detected by passing leukapheresis products through a CellSearch system [30].

### 2.1. Automated Identification of CTC

CTC identification remains a manual process executed by trained operators. It is laborious, time consuming and highly subjective. Automated identification by image analysis algorithms would be a more suitable approach, as it eliminates the subjectivity, makes the assignment of objects as CTC highly reproducible and is fast. The caveat of automated image recognition of cells is that the images need to be acquired on the same or on very similar platforms. Differences in numerical aperture of the objective, illumination, fluorescent filters, camera characteristics and integration time used for each of the fluorochromes will have a large influence on the reported results. The algorithms developed for the CellSearch system therefore likely will not be applicable to other systems and quantitative morphological and immunological features of the CTC can only serve as a rough guideline for alternative systems.

**Table 1:** Overview of published CTC isolation technologies comparing parameters used for CTC definition and identification. Only technologies already tested with patient samples were included. The most common parameters were compared across publications, but in some cases other parameters not specified in the table heading were considered in the original publication. For more details about each column, please see main text. As an example, the first entry of the table should read: CTC definition was size based and none of the other specified parameters were taken into account; CTC objects were not identified using images therefore numerical aperture is not an applicable parameter in this case, the assay did not include blind users and it did not include a threshold number for CTC positive objects.

Technology	CTC Definition						Assay Parameters				
	EpCAM	CKs	CD45	Nucleus	Morphology	Size	Num Aperture	Blind	Threshold	Healthy Donors	Ref
**Capture by physical properties**											
Label-free DC Impedance Based Microcytometer	−	−	−	−	−	+	NA	−	−	+	[13]
Microtube Device	+	−	−	-	−	−	NM	−	−	+	[31]
ISET	−	NC	−	NC	NC	−	NM	−	−	+	[32]
Filter Based Microdevice	−	+	−	NC	NC	−	NM	−	−	+	[33]
**Capture by immunological properties**											
CellSearch	+	+	+	+	+	+	M	+	+	+	[9]
CTC-chip	+	+	+	NC	NC	NC	NM	+	−	+	[34]
HB-Chip	+	NC	+	NC	NC	NC	NM	+	+	+	[35]
MagSweeper	+	−	−	−	−	−	NM	−	−	+	[36]
**Capture by physical and immunological properties**											
GEDI-CTC	−	−	+	+	+	−	M	+	−	+	[37]
^pos^CTC-iChip	+	+	+	+	−	−	NM	−	+	+	[12]

+/−: applied/not applied to definition or assay parameter; NA: not applicable; M: mentioned; NM: not mentioned; NC: not clear.

Recently algorithms for automated identification of CTC were developed using stored images from samples from castrate resistant prostate cancer (CRPC) patients processed with the CellSearch system [38]. The image analysis algorithm identified potential CTC by object segmentation in the cytokeratin (CK) image and measured various properties of the object. Next, it automatically detected four independent features (size, CK-PE, CD45-APC and DNA-DAPI intensity) on the identified objects with the highest impact on Cox Hazard ratio (HR) of patient survival data. With these features, the algorithm created different classifiers by varying the inclusion criteria of the four different features. The results of each classifier were compared to those of manual identification and within the data set tested, many classifiers provided comparable HR for both baseline and follow up samples as the manual CellSearch CTC identification [38]. The optimal classifier (CK-PE > 50 counts, DAPI ≤ 170 counts, CD45-APC < 60 counts and size between 34–224 µm^2^) was then verified with an independent data set of CRPC patients, which again resulted in comparable HR as the manual CTC count, being, to the best of our knowledge, the first automated algorithm to reliably count CTC. In addition, using images from breast, colorectal and prostate cancer patients, manual and automated counting resulted in similar prognostic value for both methods. However, when limiting automated identification solely to morphological features of CTC, CTC size and nuclear-to-cytokeratin ratio had significant impact on survival for breast cancer patients, but not in the case of colorectal or prostate cancer patients [39]. 

The automated algorithm approach was also used to verify whether different CTC definitions could result in more significant CTC changes between baseline and follow up CTC counts. Four different automated CTC definitions and the manual one were compared for HR results among a set of CellSearch images from breast and prostate cancer patient CellSearch samples. Even with looser CTC definitions, both Overall Survival and Progression Free Survival were similar. Although looser CTC definitions might reduce the Poisson error associated with the sampling noise, they also include more false positives, which offsets the effect. Ultimately, the increase of the sample volume is needed to reduce the Poisson error [10,29,30]. Filtration based technologies can cope with larger sample volumes and in addition, explore size based differences between cells of interest and background cells. This would offer an alternative to enrichment strategies based on EpCAM, widely accepted as capturing molecule (Table 1), but described to be down regulated during Epithelial to Mesenchymal Transition (EMT) or in some cases not expressed at all. The next sections will further discuss CTC EpCAM expression and the requisites of filtration-based technologies as a suitable approach for detecting EpCAM^−^ CTC.

### 2.2. EpCAM^+^/EpCAM^−^ CTC

Relying on antigen expression to capture CTC implies that cells not expressing the antigen of interest will be missed. Giving the biologically heterogeneity of cells within a tumor, the density of any antigen will vary. This heterogeneity is also observed in CTC and as such it is expected that not all CTC will be captured in case only one antigen is targeted. The EpCAM antigen has been the prime choice target molecule in many of the technologies developed for CTC capture [2,12,34,35,36,37]. EpCAM is expressed to various degrees by many epithelial cancers, but not by all [40,41,42,43,44,45]. In renal cell cancer EpCAM expression is for example rare whereas in breast cancer the expression can be very heterogeneous. Moreover, down regulation of EpCAM is required for cancer cell invasion, which takes place during EMT [46], a process via which epithelial cells gain mesenchymal characteristics that empowers them to metastasize [47]. EMT-like changes have been reported on CTC as well [48,49,50]. Cytokeratin negative CTC from breast cancer patients have been described to express the EMT related antigens vimentin and fibronectin [51] and breast cancer patients negative for hormone receptors, high tumor grade, triple-negative disease or inflammatory breast cancer had increased probabilities for being undetectable by CellSearch, possibly due to CTC undergoing EMT [52]. In particular it was shown that the CellSearch did not detect normal-like breast cancer cells from cell lines [53] but combining CellSearch enrichment step with CD146 improved detection rates of breast cancer cell lines [54], as well as adding anti-CD49f to the immunostaining cocktail [55]. These data suggest that EpCAM^+^/EpCAM^−^ nature of metastasizing cells should be considered when designing CTC capture technologies.

### 2.3. Filtration-Based Technologies

Separation of tumor cells from blood cells by filtration has been described quite some time ago [56], more recently several commercial systems have become available for CTC enrichment by filtration [32,57,58]. Comparison of the sizes of leukocytes and CTC showed an average leukocyte diameter of about 8.1 μm, whereas CTC were usually larger, with an average size of 10.7 μm in the case of prostate cancer, 11 μm in colorectal cancer and 13.1 μm in breast cancer [14,39]. This difference in size indeed can be explored by technologies based on filtration. However, CTC recovery is not exclusively dependent on cell size, but also dependent on deformability and nucleus size [14]. Furthermore, fixation, for instance, increases the apparent viscosity of a cell which increases the pressure needed to pass a cell through the same pore size [59]. Also different fixatives will impact apparent viscosity differently: paraformaldehyde is a stronger fixative compared to CellSave (the proprietary fixative in ethylenediaminetetraacetic acid (EDTA) blood draw tubes used in combination with CellSearch), therefore recovery of paraformaldehyde fixed cells is lower than cells collected and fixed in CellSave tubes [59]. Furthermore, some fixatives result in the formation of precipitates, which in the case of blood will yield large aggregates of serum proteins and if used in combination with filters, will result in clogging [32]. Filters should also possess a set of characteristics that make them usable for these systems. For instance, track etch filters do not have a flat surface making the imaging process very difficult. On the other hand, TEM grids proved to be too porous and it was not possible to distinguish adjacent cells from each other [14]. Therefore filter requisites should include (i) no-reactivity with the sample (ii) maintenance of planar form during filtration and imaging and (iii) pores sufficiently separated to facilitate discrimination of cells. Regarding the number of pores, surprisingly, this is important in terms of leukocyte retention, but not important for CTC recovery, as long as the number of pores is at least equal to the number of CTC present in the sample. However the smaller the pore size, the higher the pressure needed to filter. All experiments should be conducted at a pressure lower than the critical pressure of CTC, to prevent CTC to be pulled in through the pores.

Filtration based technologies have been tested with patient blood samples diagnosed with different types of metastatic cancers and proved valuable. For instance, using polycarbonate track etch membranes with 8 µm sized pores it was possible to recover tumor liver cells from hepatocellular carcinoma patients, while healthy volunteers and chronic hepatitis patients scored negative [33]. In some cases, filtration also showed higher capture rate when compared to the CellSearch, but because CTC definitions were different for the different technologies, those with less strict definitions are more likely to score higher [32,60]. Also no data has been reported on the clinical relevance of the reported differences [60,61]. For instance, 210 blood samples from NSCLC patients were subjected to filtration (isolation by size of epithelial tumors cells, ISET) and the CellSearch assay [61]. ISET detected CTC in 104 patients whereas CellSearch only detected CTC in 82 patients [61]. On average, per patient, ISET also detected more cells than the CellSearch. However, with the ISET method, CTC were considered cells positively stained for several CKs and anti-vimentin, whereas the CellSearch requires negativity for CD45 staining, presence of nucleus, a certain size range and a cell-shape morphology, in addition to CKs staining. Nonetheless both methods correlated with Disease Free Survival. Similarly, a portable microfilter device reported higher capturing rates than CellSearch, but again only CKs and DAPI staining were considered (in addition to PSA for prostate cancer patients) in the CTC definition [32].

**Figure 1:**
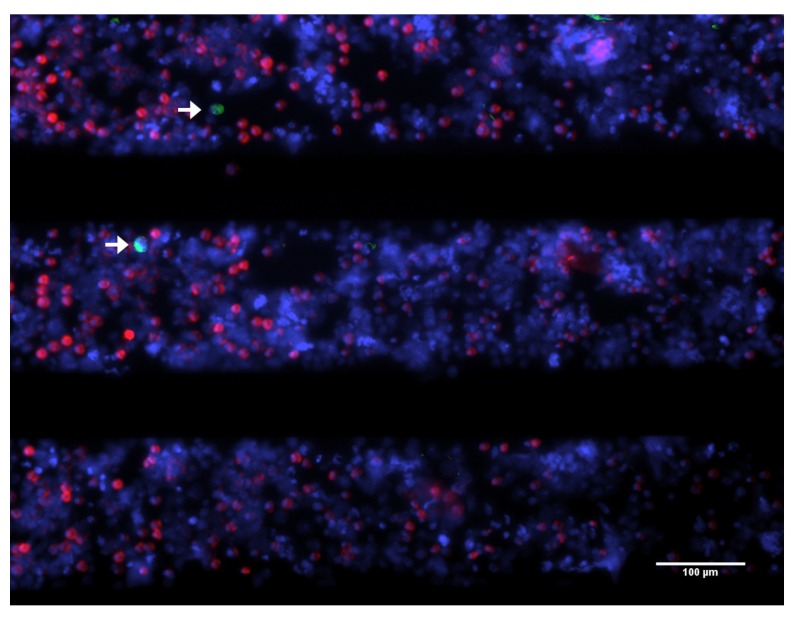
Tumor cells from CellSearch waste captured on microsieve. Blood from a healthy donor was spiked with cells from the bladder cancer cell lines, T24, that expresses low and high EpCAM density and passed through the CellTracks Autoprep. The resultant blood waste was filtered through a microsieve, showed in this image, stained for CKs (green), CD45 (purple) and nucleus (red). White arrows point towards captured T24 cells from the waste. Image taken on a fluorescence microscope with a 10× (0.45NA) objective.

To enumerate CTC not detected by the CellSearch and further understand their biological properties, our laboratory constructed a device that collects the blood discarded by the CellSearch system after immunomagnetic selection of EpCAM^+^ CTC [62]. By filtering the CellTracks Autoprep waste at a constant pressure of 100 mbar through a 5μm pore sized silicon nitride filter (Vycap, Deventer, The Netherlands) the presence of EpCAM^+^ and EpCAM^−^ CTC can be investigated. In addition the cytokeratin antibody cocktail used to identify the CTC can be expanded to investigate the presence of CTC that do not express the CKs used in the CellSearch kit. Figure 1 shows an example of a blood sample from a healthy donor spiked with cells from the bladder cell lines T24, with low EpCAM expression, processed with the CellSearch of which the blood waste was passed through a 5 μm pore sized silicon nitride filter. 

Systematic evaluation of blood samples from patients with different carcinomas will teach which CTC are present and by following the patients we can learn what the relevance is of the various CTC subpopulations. Still with this approach we will not know whether any smaller EpCAM^−^ CTC were present in the samples. To answer these questions, only a better understanding of CTC biology can help us tuning technologies to capture a real CTC, by integrating a reliable CTC definition with the technology.

## 3. Biological Properties of CTC

Morphology of CTC within and between patients is very heterogeneous [9,31,63,64] suggesting also extensive differences in their biological properties. Although many CTC are shed from the tumor(s) into the circulation, only a few will survive, successfully colonize in distant organs, promote the formation of blood vessels and consequently develop into new metastasis. The viability and proliferative state of these cells will determine to a great extent whether they have the ability to succeed.

### 3.1. Viability

**Figure 2:**
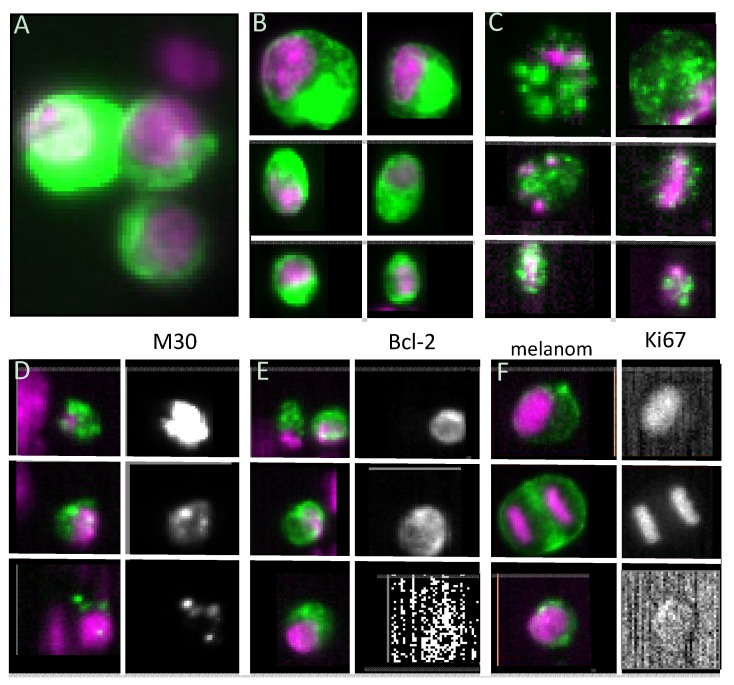
CTC heterogeneity. In all panels except for F, green represents CKs 8, 18 and 19 and purple the nuclear staining. (**A**) Cluster of three large CTC; (**B**) Individual CTC varying in size and shape; (**C**) CTC varying in size and shape exhibiting distinct punctuation of the CK staining; (**D**) CTC stained for CKs and nuclei (left) and staining of cleaved CK18 identified through M30 staining (right). Note the association between the punctuated CK staining and the M30 staining; (**E**) CTC stained for CKs and nuclei (left) and bcl-2 staining (right). Notice that only one of the CTC on the top row exhibits punctuated CK staining; (**F**) Left column showing overlay of CTC from a melanoma patient, staining of HMW-MAA (green) and nuclei (purple) and on the right column Ki67 staining.

An example of the aforementioned intra-patient heterogeneity is illustrated in Figure 2 panels A, B and C. Panel A shows a cluster of three large tumor cells that appear quite healthy and raises the question how they could have passed the peripheral vascular bed. Panel B shows six “intact” tumor cells varying in size and shape and panel C shows six “damaged” CTC varying in size and showing a distinct punctuation of the CK staining. These observations lead the hypothesis that these cells were undergoing apoptosis. This indeed appeared to be the case as this distinct cytokeratin pattern was associated with the presence of the M30 epitope present only on caspase-cleaved CK18 [64,65]. This is illustrated in panel D for three cells with punctuated CK: the panels on the left show the overlay of the CKs 8, 18, 19 FITC (green) and nucleus (purple) and the black and white images on the right the M30 staining showing the presence of the M30 epitope in these “CK containing granules”. In contrast with M30, the expression of the anti-apoptotic B-cell lymphoma protein 2 (bcl-2) suggest a viable cell resistant to apoptosis and its expression might also predict response to selected endocrine and chemotherapies [66,67]. In panel E images of bcl-2 expression of CTC is shown: on top two CTC of a breast cancer patient are shown, one of them expressing bcl-2 whereas the other does not. The image in the middle shows a CTC clearly staining with bcl-2 whereas the bottom images show a CTC that is not expressing bcl-2. The expression of M30 and bcl-2 in CTC is quite heterogeneous and whether or not this has any clinical utility has yet to be determined [68].

Analysis of M30 expression in CTC from 10 prostate cancer patients showed remarkable intra and inter-patient variability [66]. The intact proportion of CTC among patients ranged from 3% to 76% and the majority of CTC events were classified as CTC fragments [66]. In another study, among 102 Small Cell Lung Cancer (SCLC) patients, apoptotic CTC were detected in 44 (43%) and their proportion to intact CTC varied from 0.2% to 20% [6]. Among hepatocellular carcinoma though, the incidence of patients with apoptotic CTC was 23% and the ratio of apoptotic CTC to total CTC number was 8.3% [69], lower when compared to other studies [65,66,70,71,72]. Interestingly, within clustered CTC (termed as circulating tumor microemboli, CTM) none of the cells presented an apoptotic nuclear morphology and moreover, bcl-2 was detected in CTC and CTM in 18 of 30 patients [6]. In contrast to these values, the mean viability of the CTC-chip (Table 1) captured cells was approximately 98%, determined by assessing cell membrane integrity in four samples obtained from lung and prostate cancer patients [34]. Also recently, Kallergi *et al*. reported higher incidence of apoptotic CTC in early breast cancer patients than in metastatic ones and although the numbers of apoptotic CTC were reduced after treatment, no statistical data was presented on the later results [73]. Numbers of apoptotic disseminated tumor cells (DTC) in the bone marrow were also higher in breast cancer patients with stable or remissive disease than in the patients with progressive disease, 3 to 4 weeks after therapy, but a comparison between these and values before therapy (basal) among the three groups of patients was not presented, but still suggesting that high M30 levels might be associated with a favorable response to therapy [74]. On the contrary, the mean values of M30 blood serum levels were elevated in lung cancer patients, consistently at 24 and 48 hours after chemotherapy. Moreover elevated basal levels of M30 were associated with increased risk of death [75].

Besides lacking a common message, the studies mentioned above also rely on different methods to measure apoptosis, stressing the need for more controlled studies to ascertain the clinical utility of apoptotic CTC. Nonetheless we can be sure that apoptotic CTC will not be able to form distant metastasis and the ability to extract information is reduced as for example no fluorescence *in-situ* hybridization can be performed on these cells [76]. Still their presence is associated with poor prognosis and as the frequency of these apoptotic cells and bodies can be substantially higher they may be used to monitor therapy [63].

### 3.2. Proliferative Capacity

The potential of a CTC to proliferate is a requisite for their ability to colonize in distant organs. Panel F of Figure 2 shows CTC in a melanoma patient captured by expression of CD146 and stained for anti-high molecular weight melanoma-associated antigen (HMW-MAA). HMW-MAA is expressed in a large percentage of melanoma lesions with restricted distribution in normal tissue [77]. The image at the top shows a CTC expressing Ki67, an universally expressed antigen in proliferating cells but absent in quiescent ones [78]. The CTC in the middle image is clearly in the midst of cell division and verified by its Ki67 staining of both nuclei. The CTC of the same patient in the bottom image does not stain with Ki67 illustrating the diversity of proliferating stages of CTC. Reports on Ki67 expression in nine breast cancer patients with CTC showed that none of the CTC expressed Ki67 [79] whereas in another study ~25% of the CTC in breast cancer patients expressed Ki67 [73]. In SCLC patients both apoptotic and proliferating CTC were detected, but confined to the solitary cells and not detected in the tumor microemboli CTM [5]. The number of reports describing the apoptotic and proliferative status of CTC are still too scarce to make definitive statements and what, if any, effect does therapy have on the proliferative state of CTC surely will need further investigation.

### 3.3. Stem Cell-like Properties

Whereas viability and proliferative state of CTC will partially determine their capacity to successfully extravasate and form metastasis, other identity traits are crucial to the process also. It has been proposed that CTC contain the tumor stem cells disseminated from the primary tumor to the distant metastatic sites [80]. This is supported by the fact that within human breast cancer primary tumors, a subpopulation of cells expressing CD44^+^CD24^−/low^, having stem cell characteristics, were able to form tumors when only 200 of these cells were implanted in non-obese diabetic/severe combined immunodeficient (NOD/SCID) mice [81]. However tumors did not form when 20,000 cells isolated from the same tumor but not expressing the CD44^+^CD24^−/low^ phenotype were implanted in the mice. Furthermore, this subpopulation expressed the stem cell marker Oct-4 when cultured in vitro [82]. Also among DTC detected in the bone marrow of early breast cancer patients, 72% expressed the putative stem cell phenotype, CD44^+^CD24^−^ [83]. 

Studies with CTC revealed subpopulations with stem cell-like phenotypes. In a study comprising 30 metastatic breast cancer patients, it was shown that 80% of the patients had the CK^+^/CD44^+^/CD24^−/low^ phenotype among their CTC and the mean prevalence per patient was 35.3% [84]. The authors also observed the existence of a less common observed population of aldehyde dehydrogenase 1 (ALDH1)^high^/CD24^−/low^. ALDH1 has been associated with poor prognosis in different forms of breast cancer [85,86] and it has been shown that both normal and malignant stem and progenitor cells display high ALDH activity [87,88,89]. Therefore, the ALDH1 expressing CTC among the CD24^−/low^ phenotype may further distinguish a subpopulation of higher metastatic potential. Accordingly, breast cancer cells displaying high ALDH activity and the CD44^+^CD24^−^ phenotype were highly tumorogenic compared to cells that did not display the CD44^+^CD24^−^ pattern [81].

Recently CTC were identified in blood samples of 82 hepatocellular carcinoma patients in a cohort consisting of 123 [69]. EpCAM^+^ CTC were isolated using the CellSearch profile kit, cytospined and immunostained for stem cell-like markers according to a random separation of the blood samples for different stainings. CTC were found positive for stem cell-like markers such as CD133, ATP-binding cassette sub-family G member 2 (ABCG2) and β-catenin. However, none of the CTC was positive for CD90, at least in the group assigned to that staining. It would be interesting to know the separate tumorogenic potential of these CTC subpopulations, as it was shown for cells from primary tumors. For instance, CD133^+^ cells isolated from human brain tumors were capable of initiating tumor formation in NOD/SCID mouse brains whereas the CD133^−^ fraction could not, even when a thousand times more cells were implanted [90]. Moreover co-expression of CD133 and ALDH1A1 in surgically resected lung tumors of early stage NSCLC patients was strongly associated with poor survival [91].

Major signalling pathways that characterize normal stem cells were found activated in tumor cells and CTC as well. CTC from patients with primary and metastatic breast cancer were shown to express receptors and activated signalling kinases of the EGFR/HER2/P13K/Akt pathway [92]. The authors analyzed peripheral blood mononuclear cells (PBMC) from 32 early and 16 metastatic breast cancer patients and reported that phospho PI3K/Akt were identified in a significant proportion of CTC. The pathway PTEN/Akt/mTOR has been described as one of the major pathways in the regulation of mammary stem/progenitor cells and to play a central role in the viability and maintenance of cancer stem cells in breast, promoting proliferation and the inhibition of apoptosis [93,94].

CTC with stem-cell like traits might represent a more aggressive subset of CTC. However, the clinical utility of stem cell-like phenotypes needs further investigation. Regarding ALDH1 for instance, it was still not possible to associate ALDH1 positivity with any of the studied clinopathological factors [95], although within the group of patients with CTC 46% displayed the ALDH1^+^ phenotype against 5% of the patients without CTC. In another study, it was not feasible to evaluate the clinical relevance of the expression of CD44^+^CD24^−^ or ALDH1^high^CD24^−/low^ phenotype among CTC due to the heterogeneity of the study patients population [84], again claiming the need for more controlled studies.

## 4. CTC *in Vitro* Culturing and *in Vivo* Models

The ability to expand CTC will greatly enhance the ability to study the metastatic process, explore new drugs with anti-tumor activity and test the anti-drug or drug combination that is most suitable to treat the individual patient.

### 4.1. CTC *in Vitro* culturing

**Figure 3:**
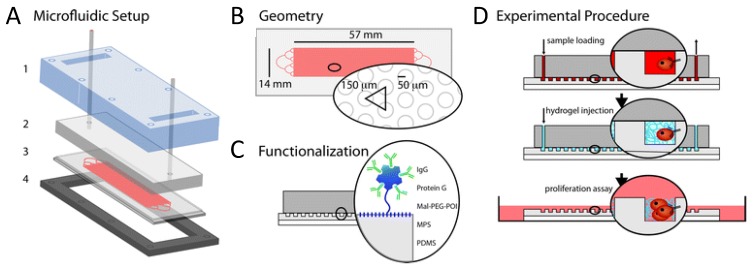
Proposed microfluidic setup by Bichsel *et al*. [97] that allows cancer cells to be transferred from a capturing microchip device to a cell culture dish. (**A**) Microfluidic setup with transparent cover (1), cover with inlet and outlet tubing (2), microstructured PDMS on a glass slide (3) and metal support frame (4); (**B**) Detail of the microstructured PDMS geometry containing pillars of 100 µm in diameter; (**C**) PDMS functionalization principle; (**D**) Schematic of sample loading in the microfluidic device and magnification of a pillar-bound cell, followed by injection of the hydrogel precursor that solidifies and encapsulates the cells. Gel-encapsulated cells are finally transferred to cell culture conditions to be grown. Reproduced from [97] with permission from The Royal Society of Chemistry. For more details, the reader is referred to the original publication.

CTC *in vitro* culture requires the cells to be viable after extraction from the blood. However most technologies depend on intracellular staining (e.g., CKs), which implies loss of cellular viability due to fixation and permeabilization methods. Some authors have presented solutions that preserve cell viability, generally at the cost of diminishing the number of molecular targets, which compromises isolation of a more heterogeneous CTC population and at the risk of isolating more hematopoietic cells. Though only tested on cancer cell lines, Takao and Takeda [96] developed a potential protocol for CTC enumeration consisting of a flow cytometer using a disposable microfluidic chip that allows sample collectability afterwards. The cancer cells (PC-9 and MCF-7) were isolated from whole blood with EpCAM-microbeads and isolated fractions labeled with an antibody targeting a different EpCAM epitope, anti-CD45, Syto9 and PI dyes (live/dead staining). They successfully gated live cancer cells. Repeating this protocol without the blood mixing step allowed for captured MCF-7 and PC-9 to be collected from the outlet reservoir of the chip and transferred to the culture dish with standard culture medium where they grew normally, demonstrating that the procedure is non-cytotoxic and enables capturing of live cells. However, this procedure was so far tested only with cell lines and the starting amount of cells was very high (5,000 cells), compared to patient samples that might bare a few CTC only. In those cases collection of cells from the chip will be more challenging, therefore a strategy involving less manipulation is preferable. More recently microchip assays have provided a combined capture and in situ cell growth solution. A microstructured polydimethylsiloxane (PDMS) chip containing round pillars functionalized with anti-EpCAM was tested for its capturing efficiency with PC3-9 cells spiked in blood and stably expressing mCherry fluorescent protein [97]. After capture, cells were encapsulated in the chip by injecting a polyethylene glycol (PEG) precursor that jellified inside the chip, encapsulating the cells in a three-dimensional (3D) protective environment. Afterwards, the PDMS chip containing the hydrogel and supportive glass were transferred to a Petri dish for cell culture under standard conditions. After six days, 17% of the transferred cells were alive and spheroids of about 50 μm could be observed on the chip, sustaining that the method allows manipulation of live cells from microchip to standard culture dishes and supports tumor-like cell growth. A schematic of this microfluidic chip is depicted in Figure 3, including the experimental procedure of sample loading, hydrogel encapsulation and cell growth on petri dish.

Another microfluidic device demonstrated suitability for collection of isolated cells from mice bearing tumors and their subsequent culturing for at least four passages [98]. 4T1 mouse mammary cells were implanted subcutaneously in Balb/c mice and after 2–4 weeks mice were sacrificed and whole blood collected. Blood was mixed with anti-EpCAM coated magnetic beads, passed through the microfluidic device and isolated cells were directed to the outlet channel by shifting the position of the magnet. Collected cells were plated in 96 well culture dishes, with standard culture medium. They grew normally and remained viable throughout the entire culturing period. However the identity of these cells was not clearly established, as the only enrichment molecule used was EpCAM and no clear evidence of a “CTC only” culture was given. The issue of CTC culture purity was, however, considered by Hughes and coworkers [62]. In this study, buffy coats from breast, prostate, lung and ovarian cancer patients were flown through a polyurethane microtube, functionalized with Protein G, E-selectin fused to IgG and anti-EpCAM or anti-prostate specific membrane antigen (PSMA) (only for prostate) antibodies. Bound cells were eluted with accutase and transferred to 1 well of a 96 well plate where they grew for 5 days, when they were analyzed for purity. CTC were defined as EpCAM^+^ or PSMA^+^, DAPI^+^ and having 10–25 µm in diameter cells. Purity, the ratio of CTC positive cells per DAPI^+^ cells, was on average 66% and 37.2%, depending on whether the microtube had been pre-coated before functionalization with halloysite nanotubes or not, respectively. Culture purity is essential to understand the identity of the captured population to confidently perform characterization assays and anti-tumor drug tests. As purity was only established 5 days after culturing, it could also be the case that culturing conditions enhanced proliferation of EpCAM^−^ or EpCAM^+^ cells and the purity ratios immediately after capture were higher or lower than the ones presented in the article. This would be important to know in order to optimize and adjust culturing conditions that favor the growth of CTC rather than non-CTC cells in the culture dish.

Besides microfluidic devices, there is proof of principle that filter based technologies are suitable for culturing CTC [99]. Blood derived from tumor bearing mice was filtered through two sandwiched parylene membranes containing pore sizes of 30 µm and 8 µm (top and bottom membrane respectively) spaced by a gap of 5.4 µm. Since CTC are bigger than the remaining cells found in the blood, in principle CTC will be retained by the bottom filter while unwanted cells will be filtered out. After filtration the bottom filter was transferred to a culture dish with cell growth media and the culture was maintained for at least 21 days. Although the purity of the captured and the grown cells still needs to be demonstrated, it showed that such membranes are capable of maintaining cell viability and supporting cell growth under appropriate cell culturing conditions.

In the studies mentioned so far, either tumor cell lines were used, for which optimal culture media formulations have been long established or high starting numbers of primary cells isolated from blood. However, one of the major challenges in culturing CTC will be when the patient isolated fraction is reduced to a few cells. Assuming they are viable and capable of proliferating, still their clonogenic potential must be assured by the right cocktail of growth factors and perhaps even a different substrate than the bottom of a culture dish. Recently, Zhang and coworkers used stem cell culture medium for cell growth of CD45^−^/ALDH1^+^ isolated cells from PBMC of breast cancer patients [100]. EpCAM^+^ cultures derived from the initial population did not survive beyond 14 days, however EpCAM^−^ colonies did and were further expanded in medium for epithelial cells. Out of these cultures, 3 cell lines were established. This is, to the best of our knowledge, the first report on the generation of CTC cell lines. Further characterization of these cell lines showed expression of several tumor-initiating markers, such as EMT, CKs and stemness. The use of different media possibly provided a necessary transition phase between the state of suspension in blood and culture in the dish.

Some *in vitro* models have added a dynamic dimension to the study of metastasizing cells [101,102,103,104]. For instance, a microfluidic device was designed to study the interaction between circulating cancer cells and endothelia during the mechanism of extravasation. The authors devised a system in which a chemokyne known to attract cancer cells (CXCL12) is applied onto the basal side of the endothelia. Cancer cells flow than on the apical part (MDAMB-231). They found out that treatment with that chemokine enhances adhesion of cancer cells to endothelia through expression of CXCR4 on the endhothelia independent of the expression of CXCR4 or CXCR7 on the cancer cells [101]. Such “lab on a chip” models permit the understanding of specific interactions that static in vitro cultures don’t. However, currently only animal models can provide a global physiological response to different study parameters, including drug response. In the next section, we will elaborate on the current existent *in vivo* models to study CTC.

### 4.2. CTC *in Vivo* Assays Using Mouse Models

CTC collected from patients can provide valuable information about their biology, and if successful cultured *in vitro*, serve as basis for anti-tumor drug screening. However, animal models can add on the dynamics of metastasis and drug response at a physiological level. Using *in vivo* flow cytometry it was possible to track fluorescently labeled cancer cell lines when injected intravenously in mice [105]. Another study used an orthotopic cancer mouse model, instead of intravenous injection of cancer cells, by implanting tumor tissue derived from a green fluorescent protein (GFP)-expressing hepatocellular carcinoma cell line in the liver of mice [106]. CTC flow was monitored through an artery in the mouse ear and provided evidence that the number of CTC and distant early metastasis decrease after tumor resection. More recently, and in line with these data, CTC shedding into the bloodstream was monitored using tumor subcutaneous mouse model and Photoaccustic Flow Cytometry, a method combining the detection of laser-induced acoustic waves and fluorescence [107]. These experiments provided pre-clinical evidence that standard medical practices involving tumor manipulation, such as palpation during physical examination, incisional biopsy and laser therapy, enhance penetration of CTC into the bloodstream. They implanted mouse melanoma and mouse breast cancer cells, constitutively expressing GFP, in the subcutis of the mice ear or on their back. Once the tumor had developed, before, during and after manipulation they monitored the veins in the ear for acoustic waves or fluorescent signals. CTC signals were considered those above red blood cells (RBC) background, since leukocytes and endothelial cells provide low fluorescence compared to the GFP^+^ cancer cells or RBC. 

The functional capacity of potential metastasis inducing cells was tested in mouse models as well. PBMC were isolated by density gradient centrifugation using Ficoll-Paque PLUS medium and sorted via magnetic beads to enrich for EpCAM^−^/CD45^−^ and EpCAM^+^/CD45^−^ [69]. Injecting 300 of the EpCAM^+^/CD45^−^ cells in NOD/SCID mice induced tumor formation in 50% of the animals, but injection of 10,000 EpCAM^−^/CD45^−^ did not. Further enrichment within the EpCAM^+^/CD45^−^ fraction, could perhaps have resulted in a higher number of animals developing metastasis. Similar strategy, but enriching beyond EpCAM/CD45 expression, was followed by Baccelli *et al*. [108]. The authors hypothesized that bone marrow can provide a suitable environment for the engraftment of CTC, because it is already a reservoir for DTC. They showed that if at least 1,109 cells acquired after depletion of hematopoietic cells from blood of breast cancer patients, were injected in the medular cavity of the femur of mice, these animals would developed metastasis in other bones of the skeleton. Moreover, CTC, defined as PI^−^/CD45^−^/EpCAM^+^ cells, isolated by FACS after hematopoietic depletion, were further analyzed for CD44, CD47 and hepatocyte growth factor receptor (MET) expression. Eight months after injection of 6,330 of CD44^+^/CD47^+^/MET^+/−^ cells in the medular cavity of the femur of mice, bone metastasis developed. Primary tumor, patient bone metastasis and mice bone metastasis all expressed MET, which led the authors to hypothesize that metastasis inducing CTC are enriched among CD44^+^/CD47^+^/MET^+^ CTC and indeed that subpopulation correlated better with disease progression than the bulk CTC. What remains to be understood is whether the injection of such large numbers of CTC are within physiological ranges of DTC harbored in the bone marrow of patients. 

## 5. Conclusions

**Figure 4:**
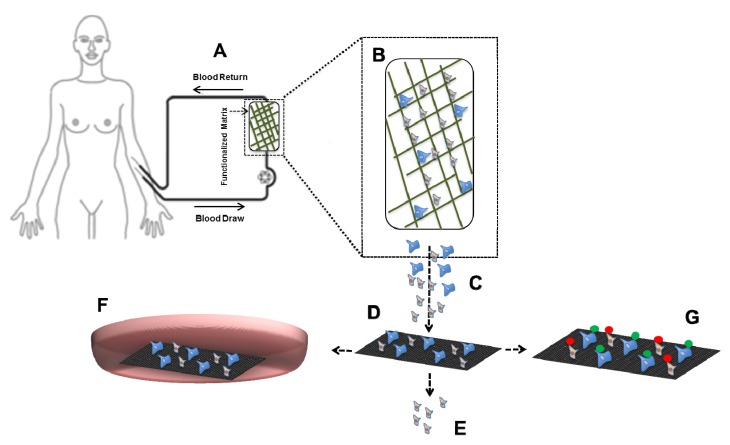
CTCTrap schematic. (**A**) Blood from a patient is circulated through a (**B**) functionalized 3D matrix (with 1 or more antibodies targeting CTC specific markers) that can withstand up to 5 L of blood flow and porous enough to prevent clogging while still able to retain cells; (**C**) Eluate of retained cells will be (**D**) filtered through 1–5 µm pores to reduce (**E**) hematopoietic background and retain the cells of interest; (**F**) Alternatively, retained cells can also be transferred to a culture dish containing appropriate growth conditions that allow selective expansion of CTC. Expanded cells can be further used to test anti-tumor drugs; (**G**) Retained cells will be further immunostained to discriminate CTC from non-CTC objects and enumerate them for patient prognosis.

Technologies for CTC capture explore the properties that distinguish them from the hematopoietic background. CTC capture targeting EpCAM, the gold standard so far, will need to be expanded to explore the full potential of CTC. Next to CTC capture based on antigen expression, CTC can be obtained by either depletion of all the blood components or separation based on differences in physical properties. The heterogeneity of antigen expression and physical properties of tumor cells makes it quite a challenge to identify the optimal approach for CTC selection and identification. Besides CTC enumeration and assessment of their protein and gene expression to monitor and select therapy, expansion of CTC could facilitate screening of actual response to anti-tumor drugs and choose the best therapeutic agent for each patient. CTC must however be viable and retain their clonogenic potential when transferred to the culture dish. In Figure 4, we propose an alternative for CTC enumeration based on whole blood inspection, currently being explored by the European consortium CTCTrap [109]. It combines immuno-capture and size based separation for CTC enumeration and characterization including the ability to culture the CTC. In our view, such approach will provide a more effective and complete platform to capture, enumerate and characterize CTC than current technologies and enable their detection in all patients with known and unknown metastatic disease.
